# Under the Skin of a Lion: Unique Evidence of Upper Paleolithic Exploitation and Use of Cave Lion (Panthera spelaea) from the Lower Gallery of La Garma (Spain)

**DOI:** 10.1371/journal.pone.0305109

**Published:** 2024-06-03

**Authors:** Marián Cueto, Edgard Camarós, Pedro Castaños, Roberto Ontañón, Pablo Arias

The following information is missing from the Acknowledgement section: The present research has been conducted under the doctorate programme on Prehistoric Archaeology at the Autonomous University of Barcelona.
